# The Effect of Infertility on the Liver Structure, Endocrinology, and Gene Network in Japanese Flounder

**DOI:** 10.3390/ani11040936

**Published:** 2021-03-25

**Authors:** Qi Mang, Jilun Hou, Tian Han, Guixing Wang, Yufen Wang, Yufeng Liu, Yuqin Ren, Yaxian Zhao, Zhongwei He, Xiaoyan Zhang

**Affiliations:** 1 Key Laboratory of Aquatic Genomics, Ministry of Agriculture, Beijing 100141, China; mangq@cafs.ac.cn (Q.M.); jilunhou@126.com (J.H.); 2 Chinese Academy of Fishery Sciences, Beijing 100141, China; 3 Beidaihe Central Experiment Station, Chinese Academy of Fishery Sciences, Qinhuangdao 066100, China; ht940309@163.com (T.H.); 13903343053@163.com (G.W.); wangyf-8000@163.com (Y.W.); bdhsyzlyf@163.com (Y.L.); renyuqin123@sina.com (Y.R.); xiaoxian20080225@126.com (Y.Z.); 15232330163@163.com (Z.H.)

**Keywords:** *Paralichthys olivaceus*, doubled haploid, infertile, vitellogenin, ceRNA

## Abstract

**Simple Summary:**

Infertility is a serious disorder that is characterized by the absence of fecundity in an individual. Not only does infertility in human beings affect reproduction, it also impacts hormonal secretion, metabolism, and the immune system. The doubled haploid (DH) that is induced by artificial mitogynogenesis or androgenesis in fish often results in infertility. The causes of infertility and its effects on other organs in the DH Japanese flounder (*Paralichthys olivaceus*) have not yet been established. Due to many aspects, the reproductive biology of this species is largely unknown or incomplete. Here, we compared the liver structure and hormone levels between fertile and infertile Japanese flounders, constructed ceRNA networks, and performed the integrated analysis of transcriptomics and proteomics. We concluded that gonadal infertility is associated with not only changes in histological structure and hormone secretion, but also changes in metabolism, immunity, and signal transduction networks in the liver. We also identified and characterized the differently expressed non-protein coding transcripts that are involved in liver functions in response to infertility. This study expanded our understanding of the molecular mechanisms of infertility on fish.

**Abstract:**

The liver can synthesize vitellogenin, the precursor of vitellin, which is needed for oocyte development and maturation. Here, we investigated the effects of infertility on liver structure, hormone regulation, and gene and protein networks in Japanese flounder (*Paralichthys olivaceus*). Results showed that the liver of infertile fish had fewer vacuoles and significantly lower serum vitellogenin (VTG) level than in liver of fertile fish. Whole transcriptomics analysis between infertile and fertile groups identified 2076 significantly differentially expressed (DE) mRNAs, 431 DE lncRNAs, 265 DE circRNAs, and 53 DE miRNAs. Proteomics analysis identified 838 DE proteins. Integrated analysis of whole transcriptomics and proteomics revealed 60 significantly DE genes and proteins associated with metabolism, immunity, signal transduction, and steroid biosynthesis. Moreover, non-coding RNA (miRNAs, circRNA, and lncRNA) transcripts involved in metabolism, immunity, and signal transduction in infertile liver were identified. In conclusion, this study shows that gonadal infertility is associated with not only changes in histological structure and hormone secretion but also changes in metabolism, immunity, and signal transduction networks in the liver. These results provide valuable information concerning the mechanism underlying infertility in fish.

## 1. Introduction

Infertility is a serious disorder that is characterized by the absence of fecundity in an individual. This condition affects about 10% of all human families worldwide and, probably, more in the developing countries [1]. Not only does infertility in human beings affect reproduction but it also impacts hormonal secretion, metabolism, and the immune system [2,3]. 

The liver is a crucial organ that regulates many important functions, including metabolism, immune system, storage, and secretion [4,5]. In addition, it is the primary organ for vitellogenin (VTG) synthesis. Vitellogenin is a precursor protein for egg yolk vitellin, that serves as a molecular vehicle in the transportation of protein, fat, and vitamins to oogonia. The levels of VTG in serum are correlated with gonadal development [6,7]. As the organ for VTG synthesis, the role of the liver in reproduction cannot be ignored.

Japanese flounder, *Paralichthys olivaceus*, is an economically important fish species for marine culture in China, Japan, and Korea. In the recent 20 years, mass production of doubled haploid (DH) in Japanese flounder were successfully fulfilled [8]. While, DH that is induced by artificial mitogynogenesis or androgenesis in fish often results in infertility [9,10]. This condition was also characterized by varying levels of secreted hormones, obesity, and dysgenesis of the ovary [11,12]. These infertile DH individuals were good source material for research into the effects of infertility on the body, especially for identifying gene and protein network that are indispensable for reproduction in fish. 

Advances in high-throughput techniques have improved the efficiency of study outcomes on the regulatory mechanisms at the transcriptional and post-transcriptional levels. By sequencing RNA transcripts, the functions of some non-coding RNAs such as microRNAs (miRNAs), long non-coding RNAs (lncRNAs), and circular RNAs (circRNAs) have been found [13]. According to the theory of competing endogenous RNA (ceRNA), circRNAs negatively regulate the activity of miRNAs as miRNA sponges, and lncRNAs compete with mRNAs by binding to miRNAs [14]. Therefore, a better explanation for phenotypes and establishment of regulatory networks can be achieved by applying whole-transcriptome approaches that include mRNA, miRNA, lncRNA, and circRNA profiling. Comprehensive proteomic analysis can be done by label-free quantitative proteomic methods based on LC-MS/MS [15]. In fish, label-free quantitative proteomics have been used in the studies of development, immune system, stress responses, and toxicity [16], with promising results.

The causes of infertility and its effects on other organs in the DH Japanese flounder have not yet been established. This is because many aspects of the reproductive biology of this species are largely unknown or incomplete. In this study, we compared the liver structure and hormone levels between fertile and infertile Japanese flounders and, constructed ceRNA networks of whole liver transcriptome. Through whole-transcriptome sequencing and label-free quantitative proteomic technologies, we performed the integrated analysis of transcriptomics and proteomics. Our findings elucidate on the mechanisms underlying infertility in fish. 

## 2. Materials and Methods

### 2.1. Ethical Statement

The use of the Japanese flounders in this study was done in accordance with the Guidelines for Care and Use of Laboratory Animals by the Chinese Association for Laboratory Animal Sciences (No. 2011–2). The experiments were approved by the Animal Care and Use Committee of Beidaihe Central Experiment Station, Chinese Academy of Fishery Sciences.

### 2.2. Fish Sampling 

The meiogynogenetic and DH Japanese flounders used in this study were induced in 2013 according to standard induction method as previously described [17]. From the year 2016 to 2018, the fertility of the induced fish was checked manually every year. The meiogynogenetic fish that spawned eggs every year were identified as the fertile group; while the DHs that exhibited a flat abdomen in each breeding season and did not spawn any egg were identified as the infertile group. 

The gonads of the infertile fish were developed during the early vitellogenesis stages in the breeding season. To eliminate the effect of the gonad development stage on hormone secretion, three fertile fish whose gonads were developed during the early vitellogenesis stages were selected as the negative controls for hormonal assay and liver histology. 

Three fish from each group were anaesthetized with MS-222 at 60 mg/L, the blood of each fish was aseptically obtained from the caudal vein with a disposable sterile syringe and immediately transferred into 2 mL centrifuge tubes. To isolate the plasma, the blood was centrifuged at 2000 rpm for 10 min. Purified plasma was stored at −20 °C until use.

For whole transcriptomic and proteomic analysis, the livers were dissected, immediately frozen in liquid nitrogen, and stored at −80 °C until use. For histological analysis, the livers and gonads from the infertile, fertile, and negative controls were fixed in Bouin’s solution and transferred into 75% ethanol after 24 h of fixation.

### 2.3. Histological Analysis and Hormonal Assays 

For histological analysis, the fixed livers were dehydrated in an ethanol series, embedded in paraffin, sectioned into 8 μm slices, and stained with Harris’ hematoxylin and eosin. Slides were observed under a Leica DM4000 microscope and photographs were taken using a Leica DFC490 CCD (Leica Microsystems, Wetzlar, Germany).

The plasma concentrations of 17β-estradiol (E2), androgen (T), vitellogenin (VTG), luteinizing hormone (LH), and follicle-stimulating hormone (FSH) were measured using fish-specific ELISA kits (Huding Co., Ltd., Shanghai, China), according to the manufactures’ protocols. ANOVA followed by LSD multiple comparisons (*p* < 0.05) was conducted to compare sex steroid concentrations.

### 2.4. RNA Extraction, Library Construction, and Sequencing

Total RNAs from liver samples of the infertile and fertile group were extracted with TRIZOL Kit (Invitrogen, Carlsbad, CA, USA), and their concentrations were assessed using the NanoVue Plus Spectrophotometer (IMPLEN, Westlake Village, CA, USA). Equal amounts of RNA from each infertile fish were pooled into one group, the same procedure was done for RNA from each fertile fish. The cDNA library of mRNA/lncRNA and circRNA and small RNA was generated, respectively, using the rRNA-depleted RNA by NEBNext Ultra Directional RNA Library Prep Kit for Illumina (NEB, Ipswich, MA, USA) and NEBNext Multiplex Small RNA Library Prep Set for Illumina (NEB, Ipswich, MA, USA.). rRNA was removed using an Epicentre Ribo-zero rRNA Kit (Epicentre, Madison, WI, USA). mRNA, lncRNA, and circRNA sequencing was performed using Illumina HiSeq^TM^ 4000, while small RNA sequencing was performed using Illumina HiSeq 2500.

### 2.5. Analysis of Protein-Coding and lncRNA Transcripts 

Raw data were subjected to quality checks through in-house perl scripts. To obtain high-quality clean reads, reads containing more than 50% of low quality (Q-value ≤ 20) bases, reads containing more than 10% of unknown nucleotides, and reads containing adapters from raw reads were removed with ng_qc (1.0). The index of the reference genome was built using bowtie2 (v2.2.8), and paired-end clean reads were aligned to the reference genome of the Japanese flounder [18] using HISAT2 (v2.0.4) [19]. The mapped reads of each sample were assembled by StringTie (v1.3.1) in a reference-based approach [20]. The CNCI (Coding-Non-Coding-Index) (2), CPC (Coding Potential Calculator) (0.9-r2), PhyloCSF (phylogenetic codon substitution frequency) (v20121028) 25, and Pfam Scan (v1.3) programs were used in the prediction of the protein-coding potential of new transcripts with default parameters. The lncRNA transcript yield was obtained at the intersection of the results without protein-coding potential. Cis- and trans-regulation analysis was used to predict the target genes for lncRNA transcripts. The expression levels of all transcripts were normalized using FPKM with the software Cuffdiff (v2.1.1). Transcripts with an FDR (false discovery rate) of <0.05 and a fold change of ≥2 were identified as significant differentially expressed (DE) protein-coding or lncRNA transcripts using Cuffdiff (v2.1.1) [21].

### 2.6. Analysis of circRNA Transcripts 

The circRNAs were detected and identified using find_circ (1.1) and CIRI2 (1.2) [22]. The Circos figure was constructed using the Circos software (v0.62-1). The raw counts were normalized using TPM [23]. The differential expression analysis of the two groups was performed using the DEGseq R package (1.12.0). The adjusted *p*-value was set as the threshold for significantly differential expression.

### 2.7. miRNA Analysis

To obtain clean reads, raw reads were further filtered by removing reads containing ploy-N, with 5′ adapter contaminants, without 3′adapter or the insert tag, containing ploy A or T or G or C and low-quality reads from raw data. To analyze the expression and distribution of the small RNA tags on the reference, they were mapped to the reference sequence by Bowtie (0.12.9) [24]. Mapped small RNA tags were searched against miRBase (20.0) to identify known miRNAs. Based on the position of their genome and hairpin structures as predicted by the miREvo (v1.1) [25] and Mirdeep2 (2.0.0.5) [26] software, novel miRNA candidates were identified. The miRNA expression level was calculated and normalized to transcripts per million (TPM). miRNAs with an adjusted *p* value < 0.05 were identified as being significant differentially expressed. Predicting the target gene of miRNA was performed by miRanda (3.3a) [27]. 

### 2.8. Functional Enrichment Analysis

Gene Ontology (GO) enrichment analysis of DE mRNAs, lncRNA, circRNAs, and miRNAs was performed by the GOseq R package (Release 2.12) [28]. The Kyoto Encyclopedia of Genes and Genomes (KEGG) pathway analysis for DE and target genes was done using the KOBAS software (v2.0) [29]. The results with a *p*-value of ≤0.05 were considered to be significantly enriched.

### 2.9. Integrated Analysis for Whole Transcriptomics 

Based on the miRNA-lncRNA transcript, miRNA-circRNA transcript, miRNA-protein-coding transcript relationships and competitive combination with miRNAs, we constructed two ceRNA networks, i.e., the lncRNA-miRNA-gene network and the circRNA-miRNA-gene network. The ceRNA theory was applied in the investigation of the functions of lncRNA and circRNA. The analysis and visualization of the interactions were performed by Cytoscape (3.4.0).

### 2.10. Validation of Differentially Expressed Genes (DEGs) by qRT-PCR

The identified DEGs were validated using qRT-PCR. Thirteen DEGs with high levels of significance were selected for qRT-PCR analysis, and 18S was used as an internal reference. The primers used in these procedures are shown in Table S1. Reactions were done in the Quantagene q255 qPCR system (KUBO TECHNOLOGY, Beijing, China). These reactions were done in triplicates. The relative expression quantities of DEGs were determined by the 2^−ΔΔCt^ method [30]. 

### 2.11. Proteomic Analysis

Liver samples in the infertile and fertile groups were ground into a powder in liquid nitrogen and lysis buffer including 50 mM Tris-HCl (pH 8), 8 M urea, and 0.2% SDS was added. The homogenate was incubated with ultrasonication on ice for 5 min and then centrifuged at 12,000× *g* for 15 min at 4 °C. The protein concentration of supernatant was determined with a Bradford assay. To this, 2 mM DTT was added and the sample was incubated at 56 °C for 1 h. Then, sufficient iodoacetic acid was added to the sample, and it was incubated for 1 h at room temperature, protecting it from light. Additionally, 4 volumes of cold acetone were then added to a sample extract and vortexed well, and the sample was placed at −20 °C for 2 h to overnight. Further, it was centrifuged and collected as pellet and washed twice with cold acetone. Finally, the pellet was dissolved by dissolution buffer containing 0.1 M triethylammonium bicarbonate (TEAB, pH 8.5) and 8 M urea [31]. Total protein concentrations were determined by the Bradford assay (Bio-Rad, Hercules, CA, USA). Equal amounts of proteins from each infertile fish were pooled into one group. Protein pooling was also done for the fertile fish. The supernatant from each pooled protein group (containing precisely 0.1 mg of protein) was digested with Trypsin Gold (Promega, Madison, WI, USA) at 37 °C for 16 h. After digestion, the peptide was desalted with C18 cartridge to remove the high urea. The desalted peptides were then dried by vacuum centrifugation.

Shotgun proteomic analyses were performed using an EASY-nLCTM 1200 UHPLC system (Thermo Fisher Scientific, San Jose, CA, USA) coupled to an Orbitrap Q Exactive HF-X mass spectrometer (Thermo Fisher Scientific, San Jose, CA, USA) operating in the data-dependent acquisition (DDA) mode. 

The resulting spectra from each fraction were searched separately against the protein database that was created using the transcriptome dataset for Japanese flounder [18] by Proteome Discoverer (2.2) for protein identification as well as for the relative protein quantification. Proteins with at least 1 unique peptide were identified at an FDR of less than 1.0% on peptide and protein levels. Proteins that were composed of similar peptides and could not be distinguished on MS/MS analysis were grouped separately as “protein groups.” Precursor quantification based on intensity was used for label-free quantification. The protein quantitation results were statistically analyzed by the Mann–Whitney U Test. The significant ratios, defined as the ratio of those proteins ≥1.2 or ratio ≤0.8, were used to screen for the differentially expressed proteins (DEPs).

GO and InterPro (IPR) analysis were performed using the InterProScan-5 program against the non-redundant protein database (including Pfam, PRINTS, ProDom, SMART, ProSiteProfiles, and PANTHER) [32]. The COG (Clusters of Orthologous Groups) and KEGG databases were used to analyze protein family and pathway. Based on the related species, the probable interacting partners were predicted using the STRING-db server (http://string.embl.de/, accessed on 15 August 2018) [33]. The enrichment pipeline was used to perform the enrichment analysis of GO, IPR, and KEGG [34].

### 2.12. Integrated Transcriptomic and Proteomic Analysis

Expression correlation analysis, data clustering analysis, GO function, and KEGG pathway analysis of DEPs and DEGs between the transcriptome and proteomic data were performed. The protein data were also used to validate and analyze transcriptomic mutations.

### 2.13. Construction of miRNA/lncRNA/circRNA and cor-DEGs-DEPs Genes Relationship Network

Based on the gene–protein and miRNA/lncRNA/circRNA–gene relationships, we constructed the miRNA/lncRNA/circRNA-cor-DEGs-DEPs genes network. Cytoscape (3.4.0) was used to analyze and visualize the interactions.

## 3. Results

### 3.1. Gonad and Liver Histology

**Figure 1:**
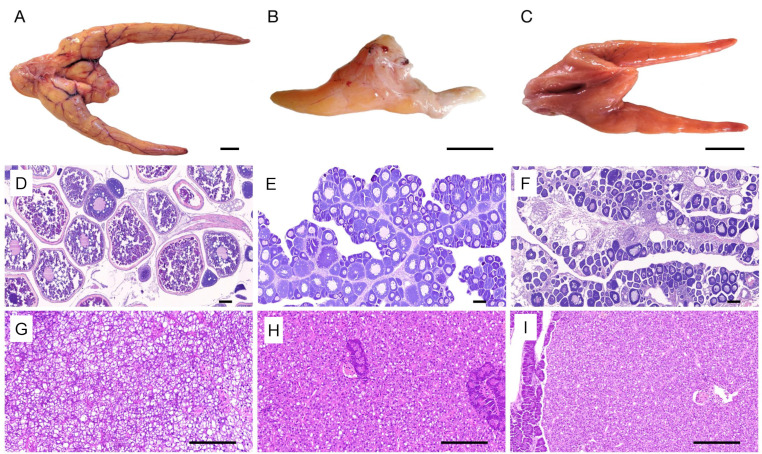
Morphological and histological characteristics of the ovary and liver in the fertile, infertile, and negative control Japanese flounder groups. (**A**) Morphological characteristics of the normal ovary of fertile group. (**B**) Morphological characteristics of the ovary of infertile group. (**C**) Morphological characteristics of the ovary of negative control group. (**D**) Histological characteristics of the ovary of the fertile group. (**E**) Histological characteristics of the ovary of the infertile group. (**F**) Histological characteristics of the ovary of the negative control group. (**G**) Histological characteristics of the liver in the fertile group. (**H**) Histological characteristics of the liver in the infertile group. (**I**) Histological characteristics of the liver in the negative control group. pv represents oocytes at Stage III, lv represents late vitellogenic oocytes, vs. represents vacuole structure of liver. Scale bars = 2 cm in (**A**–**C**), 200 µm in (**D**–**F**), and 50 µm in (**G**–**I**).

The spawning fertile group ovary appeared yellow; it presented with rich blood capillaries (Figure 1A), a transparent ovarian membrane, a large number of mature eggs near the cloaca, while histological sections indicated that it was at stage Ⅴ (Figure 1D). For the negative control group, the ovary was orange in color and presented with less blood capillaries compared to the spawning fertile group ovary, while the histological sections indicated that it was in the early vitellogenesis stage (Figure 1C,F). The ovaries of the infertile group were found to be unilateral, underdeveloped, and did not show any separated eggs during observation (Figure 1B). The histological sections indicated that the development of the ovaries of the infertile group were arrested in the early vitellogenesis stage, and as a result, no atretic follicles were detected (Figure 1E). 

The livers from the three groups exhibited round hepatocytes with a basophilic nucleus in the center, and the liver plate had clear structure and regular arrangement. The only difference was that the livers from the infertile and the negative control groups exhibited less vacuoles compared to the fertile group (Figure 1G–I). 

### 3.2. Hormonal Concentrations 

As shown in Table 1, the statistical differences in the concentrations of T and LH among the infertile group, the fertile group and the negative control groups were not significant (*p* > 0.05). For the E2 hormone, the infertile group and the fertile group were all significantly higher than negative control group (*p* < 0.05), however, it was not significantly different between the infertile group and the fertile group. The concentration of FSH was highest in the negative control group, while the concentration in infertile and fertile groups were not significantly different (*p* > 0.05). The concentration of VTG was highest in the fertile group followed by the infertile group and negative control group. 

### 3.3. Whole Transcriptomic Analysis

**Figure 2:**
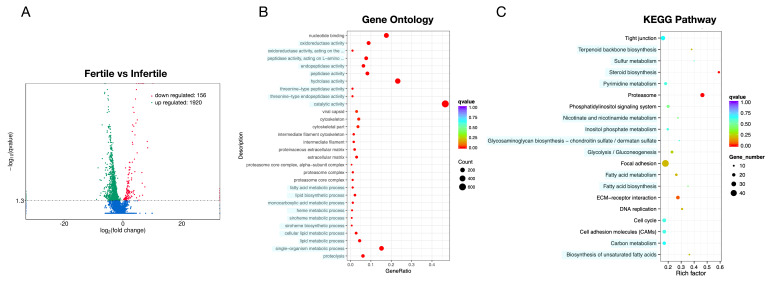
Volcano plot and Gene Ontology (GO)/pathway analysis of differentially expressed (DE) microRNAs (mRNAs) between infertile and fertile group in *Paralichthys olivaceus*. (**A**) Volcano plot of DE mRNAs. The red spots, green spots, and blue spots separately represent down-regulated, up-regulated, and undifferentiated mRNAs. (**B**) Histogram of Gene Ontology (GO) enrichment of mRNAs. (**C**) Top 20 significantly changed mRNA pathways.

A total of 2076 significantly DEGs including 1920 up-regulated and 156 down-regulated (infertile vs. fertile) were observed (Figure 2A). Functional analysis showed that the significantly different mRNAs were mainly enriched in metabolism, immunity, signal transduction, and steroid biosynthesis pathway (Figure 2B,C).

**Figure 3:**
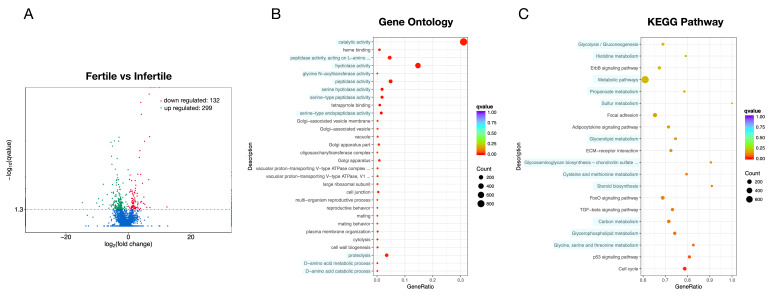
Volcano plot and GO/pathway analysis for target genes of DE long non-coding RNAs (lncRNAs) between infertile and fertile group in *Paralichthys olivaceus*. (**A**) Volcano plot for target genes of DE lncRNAs. The red spots, green spots, and blue spots separately represent down-regulated, up-regulated and undifferentiated target genes of DE lncRNAs. (**B**) Top 30 changed GOs for target genes of DE lncRNAs. (**C**) Top 20 changed pathways for target genes of DE lncRNAs.

Moreover, 431 significantly DE lncRNA transcripts were identified. Of these, 299 (69.4%) had been up-regulated, while 132 (30.6%) had been down-regulated in the infertile group (Figure 3A). The biological functions of lncRNA were predicted through its co-location with protein-coding genes, such as the lncRNA XLOC_036435 sequence, which was located on chromosome 24, 31125 bp upstream of the VTG1 mRNA and 16,990 bp upstream of the VTG2 mRNA gene (Table S2). Like the mRNA, these lncRNA co-location target genes were mainly enriched in metabolism, immunity, signal transduction, and steroid biosynthesis pathway (Figure 3B,C)

**Figure 4:**
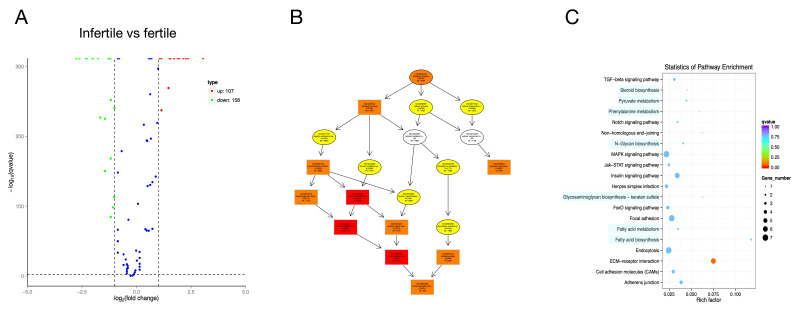
Volcano plot, Directed Acyclic Graph and pathway analysis scatter plot for target genes of DE circular RNAs (circRNAs) between infertile and fertile group in *Paralichthys olivaceus*. (**A**) Volcano plot for target genes of DE circRNAs. The red spots, green spots, and blue spots separately represent up-regulated, down-regulated, and undifferentiated target genes of DE circRNAs. (**B**) Top 10 changed GOs for target genes of DE circRNAs. (**C**) Top 20 changed pathways for target genes of DE circRNAs.

A total of 265 significantly DE circRNAs were identified, among which 158 were up-regulated, while 107 were down-regulated in the infertile group (Figure 4A). Functional analysis showed that these circRNA target genes were mainly enriched in metabolism, immunity, and signal transduction biological processes (Figure 4B,C).

**Figure 5:**
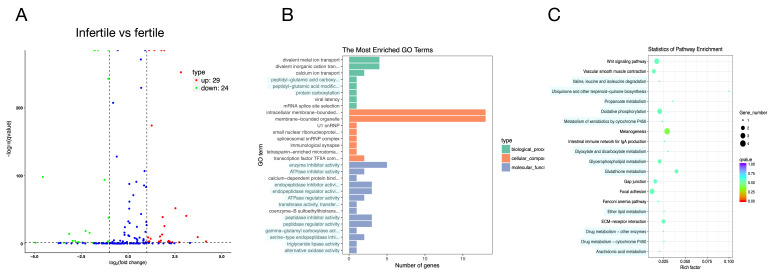
Volcano plot, GO/pathway analysis for target genes of DE miRNAs between infertile and fertile group in *Paralichthys olivaceus*. (**A**) Volcano plot for target genes of DE miRNAs. The red spots, green spots, and blue spots separately represent up-regulated, down-regulated, and undifferentiated target genes of DE miRNAs. (**B**) Histogram of Gene Ontology (GO) enrichment for target genes of DE miRNAs. (**C**) Top 20 changed pathways for target genes of DE miRNAs.

In the infertile group, 53 significantly DE miRNAs were identified, among which 27 were up-regulated, while 26 were down-regulated (Figure 5A). There were 38 novel miRNAs, and 6 known miRNAs. Functional analysis showed that these miRNA target genes were mainly enriched in metabolism, immunity, and signal transduction pathways (Figure 5B,C).

### 3.4. Construction of ceRNA Network

**Figure 6:**
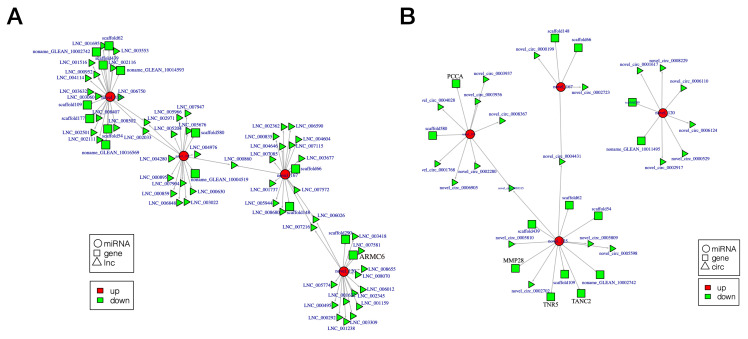
Competing endogenous RNA (ceRNA) network related to metabolism, immunity, and signal transduction between infertile and fertile group in *Paralichthys olivaceus*. (**A**) lncRNA-miRNA-gene ceRNA networks. (**B**) circRNAs-miRNA-gene ceRNA networks. Rectangle, ellipse, and triangle indicate mRNA, miRNA, and lncRNA/circRNA, respectively. Green and red indicate down-regulation and up-regulation, respectively.

Based on the data for the miRNA/lncRNA/circRNA-gene-pathway relationship network, we constructed two competing endogenous RNA (ceRNA) networks. Separately, these networks were composed of 92 lncRNAs/21 circRNAs, 14 mRNAs, and 4 common binding sites of miRNA response elements (Table S5). lncRNA/circRNA-miRNA-gene ceRNA network analysis revealed that four down-regulated miRNAs were novel (novel_103, novel_120, novel_125, and novel_167) and nine mRNAs did not have predicted gene annotations. Five target genes (*ARMC6*, *PCCA*, *TANC2*, *TNR5*, and *MMP28*) were mainly associated with metabolism, signal transduction, and immunity (Figure 6A,B). Transcriptome sequencing data have been deposited in the NCBI database (accession number: PRJNA664247).

### 3.5. Validation of DEGs by qRT-PCR

**Figure 7:**
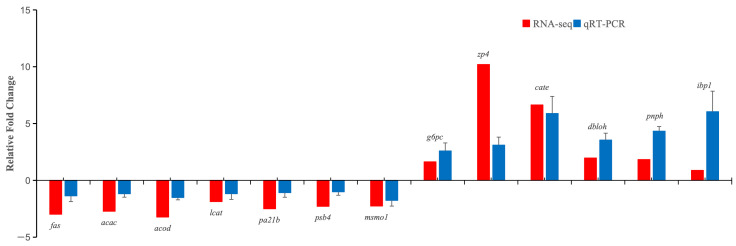
Validation of differentially expressed mRNAs using real-time quantitative PCR (RT-qPCR). Expression levels of selected differentially expressed genes (DEGs) were normalized to 18S rRNA.

To validate the results obtained by RNA-seq, 13 DEGs (*fas*, *acac*, *acod*, *lcat*, *pa21b*, *psb4*, *msmo1*, *g6pc*, *zp4*, *cate*, *dbloh*, *pnph,* and *ibp1)* were randomly selected for qRT-PCR confirmatory assays. One product was detected from each primer set and assessed by melting curve analysis. Fold changes of each gene were compared with the RNA-Seq profiles. qRT-PCR trends were consistent with RNA-Seq (Figure 7), indicating the reliability of RNA-Seq analysis.

### 3.6. Proteome Profile and Expression Differences

**Figure 8:**
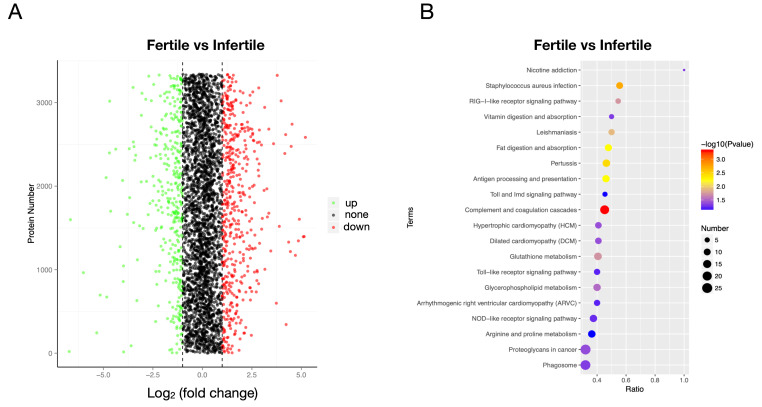
Volcano plot and pathway analysis of differentially expressed proteins (DEPs) between infertile and fertile group in *Paralichthys olivaceus*. (**A**) Volcano plot of DEPs. The red spots, green spots, and black spots separately represent down-regulated, up-regulated, and undifferentiated proteins. (**B**) Top 20 significantly changed pathways associated with DEPs.

A total of 838 DEPs were identified, among which 354 were down-regulated while 484 were up-regulated in the infertile group (Figure 8A). The most significantly different proteins such as VTG-2, were down-regulated in the infertile group. Functional analysis indicated that these different proteins were significantly enriched in the complement and coagulation cascades, fat digestion, and absorption, RIG-I-like receptor signaling pathway, and glycerophospholipid metabolism, among others (Figure 8B). 

### 3.7. Integrated Analysis of Transcriptomics and Proteomics

Changes in protein levels and their correlations with changes in the corresponding transcripts were investigated. This study found 1807 DEGs among 17,223 mRNAs and 756 DEPs among 2827 proteins. A total of 2732 correlations were found between all mRNAs and proteins. Among these correlations, 756 were between non-DEGs and DEPs, 619 were between DEGs and non-DEPs, and 258 were between DEGs and DEPs. These paired DEGS and corresponding DEPs were referred to as cor-DEGs-DEPs genes.

There were 121 cor-DEGs-DEPs genes with same down- or up-regulated trends. Sixty cor-DEGs-DEPs genes were significantly enriched in the 28 KEGG pathways (Table S3). These genes were divided into: (i) metabolic-related genes (17 cor-DEGs-DEPs genes); (ii) signal pathway-related genes (10 cor-DEGs-DEPs genes); and (iii) immunity-related genes (33 cor-DEGs-DEPs genes) (Figure S1).

### 3.8. Construction of miRNA/lncRNA/circRNA and cor-DEGs-DEPs Genes Relationship Network

**Figure 9:**
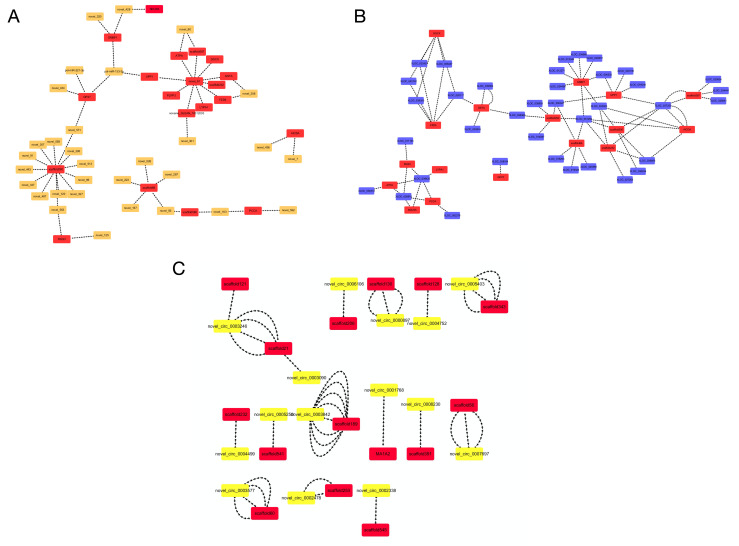
miRNA/lncRNA/circRNA and cor-DEGs-DEPs genes relationship network between infertile and fertile group in *Paralichthys olivaceus*. (**A**) miRNA and cor-DEGs-DEPs genes relationship network. (**B**) lncRNA and cor-DEGs-DEPs genes relationship network. (**C**) circRNA and cor-DEGs-DEPs genes relationship network.

We chose protein-coding and non-protein coding transcripts (miRNAs, lncRNAs, and circRNAs) predicted to be enriched in metabolism, immunity, and signal transduction pathways to construct the miRNA/lncRNA/circRNA-cor-DEGs-DEPs genes relationship network (Figure 9). Among these intersectional genes, 19 corresponded with 32 differentially expressed miRNAs, 17 corresponded with 37 differentially expressed lncRNAs, while 14 corresponded with 17 differentially expressed circRNAs. Through the interactional analysis, new miRNAs (such as novel_103, novel_120, novel_125, and novel_167) and circRNAs (such as novel_circ_0006110 and novel_circ_0008229) were associated with metabolism, immunity, and signal transductions in fish. In addition, the potential functions of some miRNAs (such as pol-miR-133-3p and pol-miR-221-3p) and lncRNAs (such as XLOC_008437, XLOC_015293, and XLOC_019323) in the liver of fish were identified.

## 4. Discussion

Reproductive success in fish depends on the biosynthesis and accumulation of VTG, which is the major yolk protein precursor [35]. The liver is the primary organ for VTG synthesis in fish [36]. Therefore, it is an essential organ for reproductive studies in fish. Fish liver also plays prominent roles in anabolism, catabolism, and in the metabolism of xenobiotics [37]. Sequencing analysis of infertile gonads had shown that genes associated with steroid hormone biosynthesis and metabolism were all down-regulated [11]. This proved that the Japanese flounder as the batch spawning fish needed more energy and nutrients during the spawning stage compared to the early vitellogenesis stage.

In our result, the genes and proteins associated with metabolism, immunity, signal transduction, and steroid biosynthesis were up-regulated in the infertile group. Fatty acid synthase (FAS) is involved in lipogenesis, and increased expression of FAS can markedly induce triglyceride deposition in body [38]. ATP-binding cassette (ABC) transporter family is an example of ATP-dependent pump, which can transport materials by utilizing the energy of ATP hydrolysis [39]. Only down-regulated pathway was glycolysis, and G6Pase and SLC were enriched in this pathway. Glucose-6-phosphatase (G6Pase) increases blood sugar concentrations by hydrolyzing glucose-6-phosphate in the liver [40], and the Solute carrier (SLC) family has been shown to be important in the transport of monosaccharides such as glucose, polyols, and other small carbon compounds across cell membranes [41]. It is, therefore, hypothesized that fertile flounders need to maintain high expression levels of G6Pase and SLC2533 in order to produce adequate energy to transport to the ovary for spawning. This showed that the liver anabolism in the infertile group is more metabolically active than catabolism, compared to the liver of the fertile group. 

VTG levels in blood increase significantly with gonad development [6]. It can be used to reflect gonadal maturity and oocyte development in aquatic animals [7,42]. While, at the same gonadal development stage, the level of VTG in the infertile group was significantly higher than the negative control group. It confirms that there is accumulation of VTG in blood of infertile group. Compared to the negative control group, E_2_ and FSH levels in the infertile group were significantly high and low, respectively. E_2_ induces VTG gene expression in fish and stimulates its release from the liver and its subsequent increase in the plasma [43], while FSH stimulates VTG uptake into vitellogenic follicles [44]. As the E2 synthetic organ, the ovary of the infertile group was unilateral, undeveloped, and had a few blood capillaries. It can be hypothesized that due to the few blood vessels in the infertile gonad, VTG uptake in these gonads was limited. This led to the accumulation of VTG in blood and HPG axis hormone secretion through E_2_-negative feedback to the hypothalamus. The outcome was the lower levels of FSH. Excess VTG in the blood would be transported to the liver and metabolized. This finding is consistent with the liver histology result. Compared to the liver of fertile fish, there were less vacuoles in the infertile fish. It was reported that the increase in vacuoles between hepatocytes in *Eleutheronema tetradactylum* was related to the increased dissolution of glycogen and lipolysis [45]. Less vacuoles between hepatocytes of infertile fish indicated that anabolism was greater than catabolism in liver of infertile group, which is also consistent with the result of genes and proteins. 

Briefly, due to less blood vessels in the infertile gonad, VTG uptake by the infertile gonad was limited. This led to the buildup of VTG in the blood. Excess VTG in the blood would be returned to the liver to be further metabolized and recycled. This changed the gene and protein networks in the liver of the infertile group. Therefore, there was an increase in metabolic and immune activities in the liver of the infertile group. This led to proteasome and DNA replication as well as their up-regulation in the infertile liver. 

Furthermore, this manuscript provides the first expression profiles of non-protein coding transcripts in the Japanese flounder liver and DE non-protein coding transcripts in response to infertility. Through functional analysis, these non-protein coding transcripts were found to have important roles in liver functions, which were mainly enriched in metabolic, immune, steroid biosynthesis, and signal pathways. miR-221-3p was found to have a proangiogenic function with the involvement of c-kit molecules and PI3K-AKT pathways [46]. gga-miR-221-5p at the peak-laying stage was significantly lower than that of the pre-laying stages, which was involved in liver lipid metabolism [47].

Integration of multi-omics can generate new knowledge that is not accessible by analysis of single datasets alone [48], the construction of ceRNA network by integrating multiple omics analyses, miRNA, and lncRNA highly relevant to ovary development in broody chickens were detected [49]. In this study, we constructed two ceRNA relationship network by integrating multiple omics analyses and subsequently detected miRNA and lncRNA/circRNA highly relevant to liver function in infertile flounders. For example, the non-protein coding transcripts miRNA novel-103, circRNA novel_circ_0003936, and novel_circ_0002280 could be related to metabolism due to their competitive relationship with the *PCAA* gene, which is involved with the metabolism pathway [50]. Similarly, miRNA novel-125, circRNA novel_circ_0005809, and novel_circ_0005598 are likely related to immune pathways due to competitively combining with the immune pathway genes *MMP28* [51], *TNR5* [52], and *TANC2* [53]. Since it was reported that lncRNA LNC_008680 had cell proliferation and growth functions [54], the transcripts that are competitively combined with it (miRNA novel-167, mRNA scaffold66, and mRNA scaffold 148) may have similar functions.

## 5. Conclusions

In summary, we investigated the effects of infertility on liver structure, hormone regulation, and gene and protein networks in Japanese flounder. We discovered that gonadal infertility is not only associated with changes in histological structure and hormone secretion but also changes in metabolism, immunity, and signal transduction networks in the liver. We also identified and characterized the DE non-protein coding transcripts that were involved in liver functions in response to infertility. This study expanded our understanding of the molecular mechanisms of infertility on fish. Such information may also have relevance to understanding reproductive disorders in other vertebrates, even humans.

## Figures and Tables

**Table 1:** Hormone concentration in infertile, fertile, and negative control Japanese flounder.

Hormones	Infertile	Fertile	Negative Control
E2 (ng/L)	72.7 ± 3.71 ^a^	75.88 ± 4.92 ^ab^	45.14 ± 5.87 ^c^
T (ng/L)	3531.95 ± 214.42 ^a^	3222.02 ± 282.10 ^a^	3599.93 ± 420.06 ^a^
LH (ng/L)	927.77 ± 126.99 ^a^	945.12 ± 151.38 ^a^	776.88 ± 204.02 ^a^
FSH (U/L)	26.22 ± 1.20 ^b^	28.78 ± 2.57 ^bc^	32.68 ± 3.48 ^a^
VTG (μg/L)	596.57 ± 59.56 ^b^	1564.13 ± 199.17 ^a^	298.43 ± 15.18 ^c^

Note: Different letters in one low indicate significant differences (*p* ≤ 0.05).

## Data Availability

All data, methods, and results of statistical analyses are reported in this paper. We welcome any specific inquiries. The NCBI accession number was PRJNA664247.

## Supplementary Materials

The following are available online at https://www.mdpi.com/article/10.3390/ani11040936/s1. Table S1: Sequence of primers used in q-PCR; Table S2: The information of long non-coding RNAs (lncRNAs); Table S3: The integrated analysis of microRNAs (mRNAs) and proteins; Table S4: The list of tools with versions and major parameter; Table S5: lncRNA/circular RNAs (circRNA)-miRNA-gene competing endogenous RNA (ceRNA) networks; Figure S1: Cluster heatmap for Kyoto Encyclopedia of Genes and Genomes (KEGG) analysis of differentially expressed genes/proteins between infertile and fertile group in *Paralichthys olivaceus*.
